# Two Sweet Sorghum (*Sorghum bicolor* L.) WRKY Transcription Factors Promote Aluminum Tolerance via the Reduction in Callose Deposition

**DOI:** 10.3390/ijms241210288

**Published:** 2023-06-17

**Authors:** Kexing Guan, Zhenming Yang, Meiqi Zhan, Meihui Zheng, Jiangfeng You, Xiangxiang Meng, He Li, Jie Gao

**Affiliations:** Jilin Province Engineering Laboratory of Plant Genetic Improvement, College of Plant Science, Jilin University, Changchun 130062, China; gkx121@163.com (K.G.); zmyang@jlu.edu.cn (Z.Y.); zhanmeiqi_523@163.com (M.Z.); z571293211@163.com (M.Z.); youjf@jlu.edu.cn (J.Y.); xiangmeng@jlu.edu.cn (X.M.)

**Keywords:** Al toxicity, WRKY, transcriptional regulation, Al tolerance genes, callose

## Abstract

Aluminum (Al) toxicity is a primary limiting factor for crop production in acidic soils. The WRKY transcription factors play important roles in regulating plant growth and stress resistance. In this study, we identified and characterized two WRKY transcription factors, SbWRKY22 and SbWRKY65, in sweet sorghum (*Sorghum bicolor* L.). Al induced the transcription of *SbWRKY22* and SbWRKY65 in the root apices of sweet sorghum. These two WRKY proteins were localized in the nucleus and exhibited transcriptional activity. SbWRKY22 showed the significant transcriptional regulation of *SbMATE*, *SbGlu1*, *SbSTAR1*, *SbSTAR2a*, and *SbSTAR2b*, which are major known Al tolerance genes in sorghum. Interestingly, SbWRKY65 had almost no effect on the aforementioned genes, but it significantly regulated the transcription of *SbWRKY22*. Therefore, it is speculated that SbWRKY65 might indirectly regulate Al-tolerance genes mediated by SbWRKY22. The heterologous expression of *SbWRKY22* and *SbWRKY65* greatly improved the Al tolerance of transgenic plants. The enhanced Al tolerance phenotype of transgenic plants is associated with reduced callose deposition in their roots. These findings suggest the existence of SbWRKY22- and SbWRKY65-mediated Al tolerance regulation pathways in sweet sorghum. This study extends our understanding of the complex regulatory mechanisms of WRKY transcription factors in response to Al toxicity.

## 1. Introduction

Nearly 30% of the total land area and more than 50% of the world’s potentially arable land is covered by acidic soils [1]. In soils with pH levels below 5.0, Al is released from clay minerals and oxides and absorbed by plant roots in the form of soluble trivalent Al ions (Al^3+^) [2]. Studies have shown that micromolar concentrations of Al^3+^ can rapidly inhibit plant root growth [3,4]; thus, Al stress has long been regarded as one of the most significant limiting factors for agricultural productivity in acidic soils [5]. Therefore, improving crop tolerance to Al has become a key concern in increasing crop yields on acidic soils.

During the long process of evolution, plants have developed two main defense mechanisms against Al toxicity. One is the exclusion mechanism that stops Al from entering root cells [6,7,8]. *SbMATE* in sorghum (*Sorghum bicolor* L.) and *HvAACT1* in barley (*Hordeum vulgare* L.), which belong to the MATE family, were the first Al resistance genes to be identified. These genes encode citric acid transporters that promote citric acid secretion to chelate Al^3+^ ions, thereby blocking their entry into the root cells [9,10]. Another is the tolerance mechanism, such as adjusting the root cell walls characteristics to change its Al-binding capacity and compartmentalizing Al after it enters the root symplast [11]. Many genes regulate plant Al resistance by altering the polysaccharide content of the cell wall. For example, the protein complex encoded by *OsSTAR1*/*OsSTAR2* in rice functions as an ATP-binding cassette transporter and could be involved in the mediation of the translocation of UDP-glucaric acid from the cytoplasm to the cell wall, thereby altering the cell wall composition and relieving Al toxicity [12]. Through regulating xyloglucan endo-transglycosylase (XET) activity, which in turn affects xyloglucan content in the cell wall and the Al-binding ability, AtXTH31 modulates Al resistance in *Arabidopsis thaliana* (L.) Heynh. [13]. Callose is a cell-wall-associated polysaccharide. Its deposition is not only a sensitive indicator of Al toxicity but is also a toxicity pathway itself [14]. The *SbGlu1* gene in sorghum, encoding β-1,3-glucanase, catalyzes callose degradation, thereby improving plant Al tolerance [14,15].

Studies on the mechanisms of Al tolerance in plants have delved into the level of transcriptional regulation. A number of transcription factors were revealed in Al resistance in plants. AtSTOP1 and OsART1 were crucial Cys_2_His_2_ zinc finger transcription factors that were first identified in *Arabidopsis* and rice, separately [16]. AtSTOP1 positively regulates the expression of several Al tolerance genes, including *AtALMT1*, *AtMATE*, and *AtALS3*, in *Arabidopsis* [16,17,18]. Similar to AtSTOP1, OsART1 also controls the expression of a large number of Al tolerance genes (e.g., *OsSTAR1*, *OsSTAR2*, *OsNrat1*, *OsFRDL4*, *OsMGT1*, *OsCDT3*, and *OsEXPA10*) [19,20,21,22]. However, both *AtSTOP1* and *OsART1* are constitutively expressed in plants, rather than as a result of Al induction. Meanwhile, in sweet sorghum, which is a variant of grain sorghum, Al stress induces the positive transcription of *SbSTOP1*. SbSTOP1 then regulates the expression of Al tolerance genes such as *SbMATE*, *SbSTAR1*, *SbSTAR2*, and *SbGlu1* [15,23,24].

The WRKY transcription factors are a class of proteins found in plants that contain one or two highly conserved WRKY domains. The WRKY domain includes an N-terminal WRKYGQK motif and a C-terminal C_2_H_2_ or C_2_HC-type zinc finger motif that can normally bind Zn^2+^ to facilitate the recognition and binding to DNA. WRKY proteins can be categorized into three groups (I, II, and III) based on the number of WRKY domains and the type of zinc finger motif (C_2_H_2_ or C_2_HC) they contain. Members of group I have two WRKY domains with C_2_H_2_-type zinc finger. Members of group II have only one WRKY domain with one C_2_H_2_-type zinc finger motif. Members of groups III have a WRKY domain with a C_2_HC-type zinc finger. The majority of WRKY family members are group II WRKY proteins [25]. WRKY transcription factors can specifically bind to the W-box *cis*-element ((T)TGAC(C/T)) in the promoter, thereby regulating the expression of W-box-containing genes and controlling numerous physiological and biochemical responses in plants [26]. Thus, WRKY transcription factors play an important role in regulating plant growth and development, as well as their resistance to biotic and abiotic stresses.

Recent research has demonstrated that WRKY transcription factors can affect plant Al tolerance ability by regulating the expression of Al resistance genes. AtWRKY46 negatively regulates the transcriptional expression of *AtALMT1*. The functional deficiency of AtWRKY46 leads to increased malic acid secretion from the root tip, which in turn reduces Al content in the root tip and enhances Al tolerance in *Arabidopsis* [27]. The direct interaction of SlWRKY42 with the W-box *cis*-acting element in the promoter region of the Al tolerance gene, *SlALMT9*, reduces the expression of *SlALMT9*, which has a negative regulatory effect on tomato’s response to Al [28]. By controlling the secretion of citric acid through *OsFRDL4*re, OsWRKY22 regulates Al tolerance in rice [29]. *AtWRKY47* overexpression in *Arabidopsis* increases Al tolerance, and a loss-of-function mutation in this gene significantly reduces Al tolerance in plants [30]. Some WRKY transcription factors exhibit self-regulation and cross-regulation properties. For example, OsWRKY42 negatively influences resistance in rice to the rice blast disease, while OsWRKY13 suppresses the expression of *OsWRKY42,* thus enhancing rice resistance to rice blast disease. *OsWRKY45* can induce self-expression and the expression of *OsWRKY13* [31]. A WRKY-like transcription factor, SbWRKY1, was identified in sorghum, which responds to Al stress by regulating the expression of *SbMATE* [32]. This indicates that under Al stress, WRKY transcription factors may play a regulatory role in sorghum. However, SbWRKY1 is a WRKY-like transcription factor with a WRKY motif (WRKYGEK) which is not identical to the typical conserved WRKY motif (WRKYGQK) in plants. In addition to SbWRKY1, it is not yet known whether there are other typical WRKY transcription factors that are involved in sorghum Al tolerance.

In this study, we identified two Al-stress-induced WRKY transcription factors, SbWRKY22 and SbWRKY65, in sweet sorghum. The purpose of this study was to investigate their functions in response to Al stress and to reveal the mechanisms that underly their efficacy in enhancing Al tolerance in sweet sorghum.

## 2. Results

### 2.1. Sequence and Structure Analysis of SbWRKY22 and SbWRKY65

**Figure 1:**
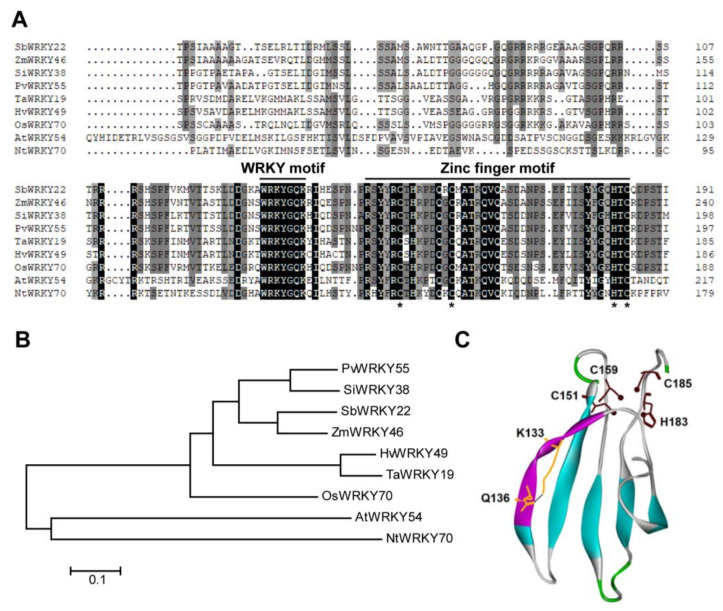
Amino acid sequence and structure analysis of SbWRKY22. (**A**) Sequence alignment of SbWRKY22 and its homologous proteins from other species, including *Zea mays* L. (ZmWRKY46, PWZ15446.1), *Setaria italica* (L.) Beauv. (SiWRKY38, XP_004958704.1), *Panicum virgatum* L. (PvWRKY55, XP_039797241.1), *Triticum aestivam* L. (TaWRKY19, XP_044327678.1), *Hordeum vulgare* L. (HvWRKY49, KAE8789191.1), *Oryza sativa* L. (OsWRKY70, XP_015646872.1), *Arabidopsis thaliana* (L.) Heynh. (AtWRKY54, AT2G40750), and *Nicotiana tabacum* L. (NtWRKY70, XP_016436463.1). Horizontal lines indicate the WRKY motif and zinc finger motif. The asterisks indicate conserved Cys_2_HisCys amino acids in zinc finger motif. (**B**) Phylogenic analysis of SbWRKY22 and the aforementioned homologous proteins. The phylogenetic tree was constructed according to the neighbor-joining method using MEGA 7. (**C**) Ribbon display model of the WRKY domain (WRKY motif and zinc finger motif) of SbWRKY22. Purple ribbon on the left: WRKY motif with the residues (yellow) forming a H-bond (black dashed line). Brown sticks at the top: Cys or His residues with S or N atoms (brown balls) bound to a Zn^2+^ ion.

**Figure 2:**
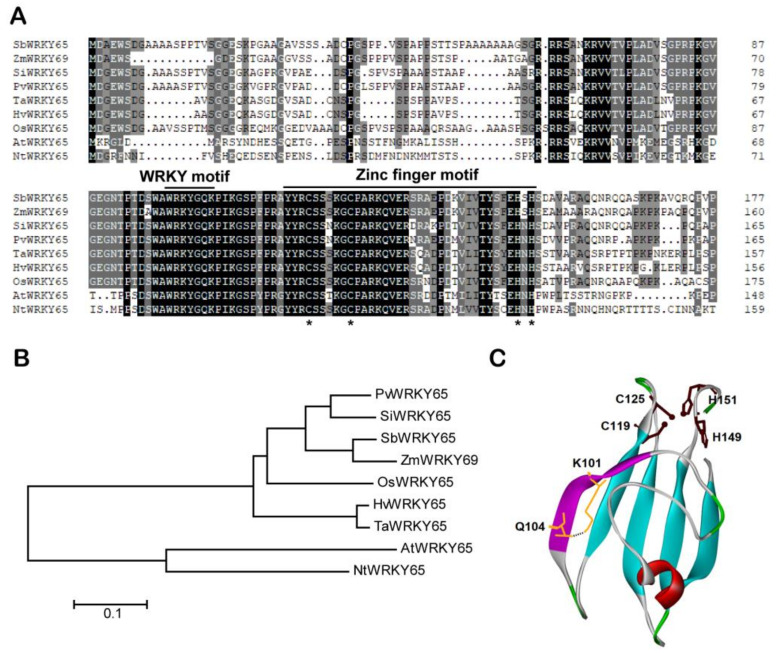
Amino acid sequence and structure analysis of SbWRKY65. (**A**) Sequence alignment of SbWRKY65 and homologous proteins from other species, including *Zea mays* L. (ZmWRKY69, ONM38198.1), *Setaria italica* (L.) Beauv. (SiWRKY65, XP_004969837.1), *Panicum virgatum* L. (PvWRKY65, XP_039813594.1), *Triticum aestivam* L. (TaWRKY65, XP_044344816.1), *Hordeum vulgare* L. (HvWRKY65, XP_044972707.1), *Oryza sativa* L. (OsWRKY65, XP_015624912.1), *Arabidopsis thaliana* (L.) Heynh. (AtWRKY65, AT1G29590), and *Nicotiana tabacum* L. (NtWRKY65, XP_016511600.1). Horizontal lines indicate the WRKY motif and zinc finger motif. The asterisks indicate conserved Cys_2_His_2_ amino acids in zinc finger motif. (**B**) Phylogenic analysis of SbWRKY65 and the aforementioned homologous proteins. The phylogenetic tree was constructed according to the neighbor-joining method using MEGA 7. (**C**) Ribbon display model of WRKY domain (WRKY motif and zinc finger motif) of SbWRKY65. Purple ribbon on the left: WRKY motif with the residues (yellow) forming a H-bond (black dashed line). Brown sticks at the top: Cys or His residues with S or N atoms (brown balls) bound to a Zn^2+^ ion.

A total of 94 putative WRKY transcription factors were retrieved from the sorghum (*Sorghum bicolor* L.) database, among which, *SbWRKY22* (Sobic.002G418500.1) and *SbWRKY65* (Sobic.003G285500.1) were found to be induced at the transcriptional level under Al stress. Therefore, the function of SbWRKY22 and SbWRKY65 was systematically examined in this study. *SbWRKY22* and *SbWRKY65* were cloned from sweet sorghum, which contained 1065 bp and 960 bp coding sequences, as well as encoding proteins with 354 and 319 amino acids, respectively. The amino acid sequence of SbWRKY22 or SbWRKY65 and their homologous proteins in other plant species were analyzed via multiple sequence alignment, and the results are shown in Figure 1 and Figure 2, respectively. Both SbWRKY22 and SbWRKY65 contain a conserved WRKY domain. The WRKY domain in SbWRKY22 contains a WRKYGQK motif and a C_2_HC-type zinc finger motif (Figure 1A), indicating that SbWRKY22 belongs to the group III WRKY transcription factor category. Phylogenetic analysis showed that SbWRKY22 was most closely related to ZmWRKY46 with 54.9% identity, while it was related to AtWRKY54 with 29.4% identity (Figure 1B). Correspondingly, the WRKY domain in SbWRKY65 also contains a WRKYGQK motif but with a C_2_H_2_-type zinc finger domain (Figure 2A), showing that SbWRKY65 belongs to the group II WRKY transcription factor category. Phylogenetic analysis showed that SbWRKY65 was most closely related to ZmWRKY69 with 76.9% identity, while it was related to AtWRKY65 with 31.7% identity (Figure 2B).

To clarify the structural properties of the WRKY domains in SbWRKY22 and SbWRKY65, molecular simulations were performed for them. The 3D structures of the WRKY domains in SbWRKY22 and SbWRKY65 (Figure 1C and Figure 2C) revealed that their zinc finger motif is highly spatially correlated with their WRKY motif. The WRKY motif contains more hydrophilic residues, and its sequence and the H-bond network it forms (only the H-bonds between K133 and Q136 in SbWRKY22 and between K85 and Q88 in SbWRKY65) directly affect the spatial orientation of the WRKY motif and the overall conformation of the zinc finger domain, which may influence the functions of SbWRKY22 and SbWRKY65 (Figure 1C and Figure 2C). On the other hand, there were some differences in the overall conformation of the WRKY domains, particularly the “finger” conformation of the zinc finger motif, between SbWRKY22 and SbWRKY65, which inevitably led to their binding to distinct DNA deep grooves, indicating that SbWRKY22 and SbWRKY65 might have different target DNA.

### 2.2. Detection of SbWRKY22 and SbWRKY65 Expression Patterns under Al Stress

**Figure 3:**
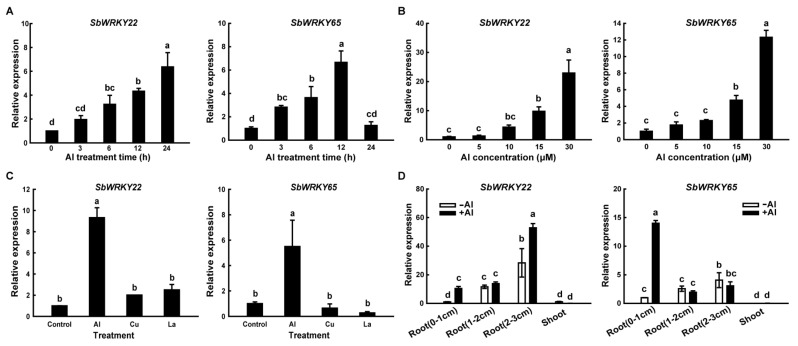
Quantitative real-time PCR analysis of *SbWRKY22* and *SbWRKY65* expression profiles. (**A**) Relative expression of *SbWRKY22* and *SbWRKY65* in sweet sorghum root apices (0–1 cm) in response to 15 μM Al stress with different treatment times. (**B**) Relative expression of *SbWRKY22* and *SbWRKY65* in root apices exposed to different Al concentrations for 12 h. (**C**) Relative expression of *SbWRKY22* and *SbWRKY65* in root apices in response to AlCl_3_ (15 μM), CuCl_2_ (0.5 μM) and LaCl_3_ (10 μM) for 12 h. (**D**) Relative expression of *SbWRKY22* and *SbWRKY65* in root apices (0–1 cm), roots (1–2 cm), roots (2–3 cm), and shoot sections in the absence (−Al) or presence (+Al, 15 μM) of Al treatment for 12 h. Data represent the means ± SD from three independent biological replicates. Columns with different letters are significantly different according to Tukey’s test (*p* < 0.05).

Real-time fluorescence quantitative polymerase chain reaction (PCR) was applied to determine the expression patterns of *SbWRKY22* and *SbWRKY65* in sweet sorghum under Al stress. Time-course studies revealed that Al stress induced a gradual increase in *SbWRKY22* expression in root apices (0–1 cm) throughout 24 h of Al treatment, with a significantly higher transcription level of *SbWRKY22* after 6 h of Al treatment than the control (0 h treatment) (Figure 3A). Likewise, the expression of *SbWRKY65* increased with increasing Al treatment duration and differed significantly from that in the control (0 h) after 3 h of Al treatment; it peaked at 12 h and then fell to a level comparable to that in the control (Figure 3A). These results showed that as compared to *SbWRKY22*, *SbWRKY65* responded to Al stress more quickly but for a shorter duration, implying that *SbWRKY65* may function during the early stage of sorghum response to Al stress, while *SbWRKY22* may function continuously in response to Al stress.

The expression of *SbWRKY22* and *SbWRKY65* in root apices was examined after treating sweet sorghum seedlings with various doses of AlCl_3_. As shown in Figure 3B, the transcript levels of both *SbWRKY22* and *SbWRKY65* were up-regulated in an AlCl_3_ concentration-dependent manner and were significantly different from those in the control samples (0 μM) when the AlCl_3_ concentration reached 15 μM. Furthermore, the induced expression of both *SbWRKY22* and *SbWRKY65* appeared to be specific to Al stress as compared to Cu and La stress (Figure 3C). Both *SbWRKY22* and *SbWRKY65* were mostly expressed in roots, rather than in the shoots, with or without Al treatment. Meanwhile, under Al stress, their expression levels were significantly improved at the root apices (0–1 cm), which is the key target site for the occurrence of Al toxicity (Figure 3D).

### 2.3. The Subcellular Localization and Transcriptional Ability of SbWRKY22 and SbWRKY65

**Figure 4:**
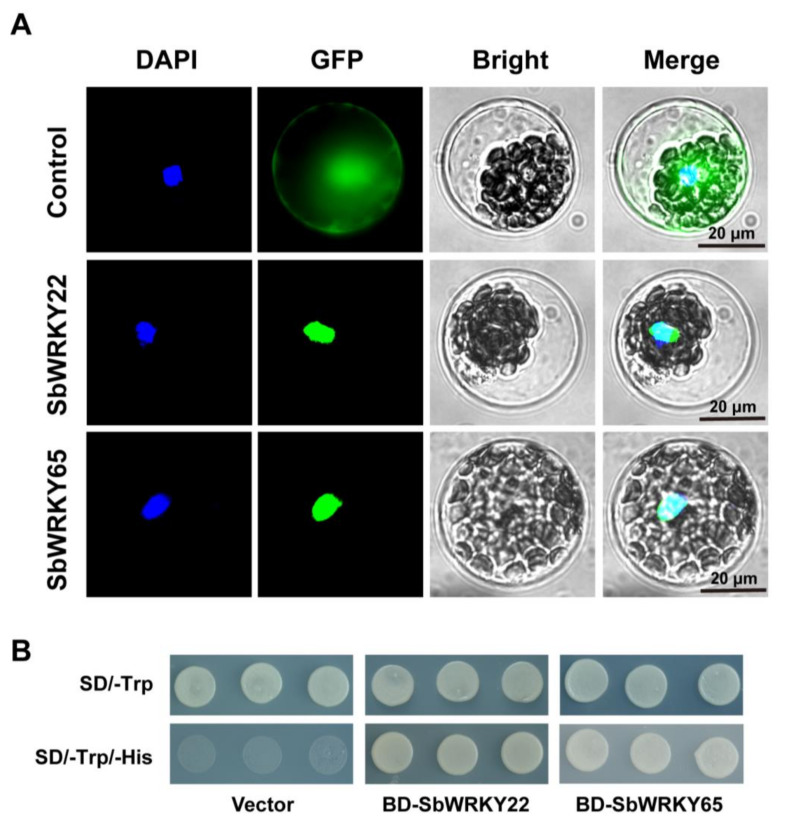
Analysis of subcellular localization and transcriptional activity of SbWRKY22 and SbWRKY65. (**A**) Subcellular localization of SbWRKY22 and SbWRKY65. Transient expression of GFP-SbWRKY22 and GFP-SbWRKY65 fusion proteins or GFP control in *Arabidopsis* protoplasts. DAPI, nuclear signal; GFP, GFP fluorescence; Bright, bright field. The scale bar is 20 μm. (**B**) Transcriptional activity of SbWRKY22 and SbWRKY65 in yeast. Y2HGold yeast strains carrying fused protein of GAL4 DNA-binding domain and SbWRKY22 (BD-SbWRKY22), SbWRKY65 (BD-SbWRKY65), or BD alone (vector) were cultured in SD/-Trp or SD/-Trp/-His medium.

To evaluate the transcriptional functions of SbWRKY22 and SbWRKY65, their subcellular localization in *Arabidopsis* protoplasts was investigated, and their transcriptional activity was examined using the yeast expression system. As shown in Figure 4A, the GFP-SbWRKY22 and GFP-SbWRKY65 fusion proteins were found to be strictly localized in the nucleus, whereas the control GFP protein was distributed throughout the cells. Moreover, the recombinant plasmids were transformed into Y2HGold yeast strains with His dystrophic markers. The results are shown in Figure 4B. In SD/-Trp media, all transfected yeast cells grew well. However, yeast cells expressing GAL4 DNA-BD alone were unable to grow normally in SD/-Trp/-His media, in contrast to yeast cells that harbored BD-SbWRKY22 and BD-SbWRKY65. These results indicate that SbWRKY22 and SbWRKY65 both have active transcriptional activity. The aforementioned results demonstrate that SbWRKY22 and SbWRKY65 feature the characteristics and effectiveness of conventional transcription factors.

### 2.4. SbWRKY22 and SbWRKY65 Overexpression in Arabidopsis Confers Aluminum Tolerance

**Figure 5:**
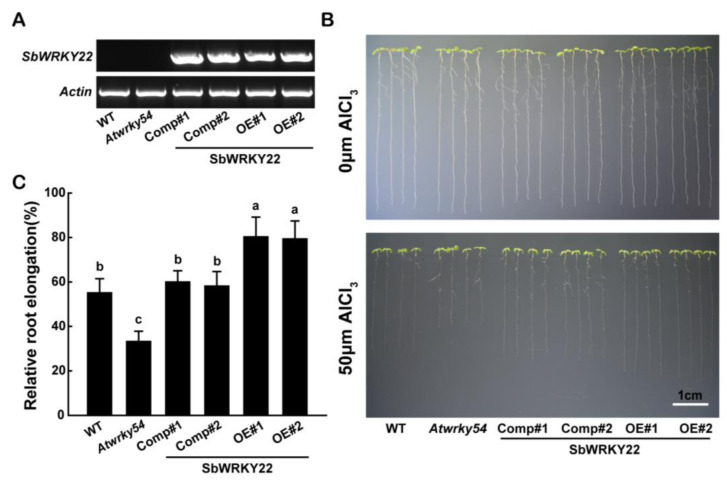
Overexpression of *SbWRKY22* in transgenic *Arabidopsis* shows improved tolerance to Al stress. (**A**) Reverse transcription polymerase chain reaction characterization of *SbWRKY22* expression in complimented lines and overexpression lines. (**B**) The Al-sensitive phenotype of WT (Col-0), *Atwrky54* mutant, and the transgenic lines. Five-day-old seedlings were precultured on a solid MS medium at pH 5.8, then transferred to a solid medium containing 1 mM CaCl_2_ and 1% (*w*/*v*) sucrose at pH 4.5, with or without 50 μM AlCl_3_ for 5 d. Scale bar, 1 cm. (**C**) Relative root elongation (root elongation with Al treatment/root elongation without Al treatment) of WT and transgenic lines. Data are shown as means with SD (*n* ≥ 20). Columns with different letters indicate significant differences between plants according to Tukey’s test (*p* < 0.05). Experiments were repeated three times.

**Figure 6:**
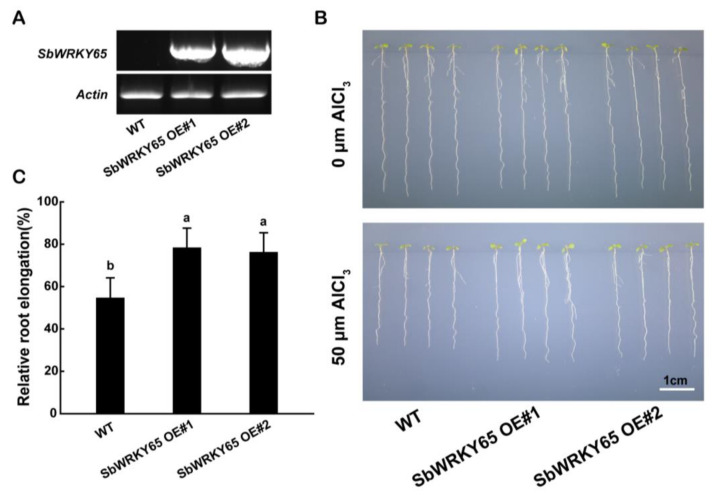
Overexpression of *SbWRKY65* in transgenic *Arabidopsis* shows improved tolerance to Al stress. (**A**) Reverse transcription polymerase chain reaction characterization of *SbWRKY65* expression in the overexpression lines. (**B**) The Al-sensitive phenotype of WT (Col-0) and the overexpression lines. Five-day-old seedlings were precultured on a solid MS medium at pH 5.8, then transferred to a solid medium containing 1 mM CaCl_2_ and 1% (*w*/*v*) sucrose at pH 4.5, with or without 50 μM AlCl_3_ for 5 d. Scale bar, 1 cm. (**C**) Relative root elongation (root elongation with Al treatment/root elongation without Al treatment) of WT and the overexpression lines. Data are shown as the means with SD (*n* ≥ 20). Columns with different letters indicate significant differences between plants according to Tukey’s test (*p* < 0.05). Experiments were repeated three times.

The rapid inhibition of plant root growth is the most apparent symptom of Al toxicity [33]. To examine the contribution of the up-regulation of SbWRKY22 and SbWRKY65 to plant root growth under Al stress, *SbWRKY22* and *SbWRKY65* were overexpressed under the control of the *CaMV 35S* promoter in *Arabidopsis*. *SbWRKY22* was also overexpressed in an *Atwrky54* mutant, as AtWRKY54 shares the highest identity with SbWRKY22 among the WRKY proteins in *Arabidopsis*. The transcript levels of *SbWRKY22* and *SbWRKY65* in the transgenic lines were examined via RT-PCR, which were then selected for phenotypic analysis (Figure 5A and Figure 6A). None of the homozygous transgenic plants differed from the wild-type (WT) control in the absence of Al, while both of them showed inhibited root elongation to varying degrees in the presence of Al (Figure 5B and Figure 6B). The relative root elongation (RRE) of WT roots was inhibited under Al treatment conditions, with an RRE of 55%. *Atwrky54*, which is sensitive to Al, had only 33% RRE, and in contrast, the two *SbWRKY22*-complemented lines greatly recovered the Al sensitivity characteristic of the *Atwrky54* mutant, with 60% and 58% RRE, respectively. All overexpression lines had significantly higher RREs than the WT, showing strong Al tolerance. The two *SbWRKY22*-overexpressing lines had 80% and 79% RRE (Figure 5C). *SbWRKY65*-overexpressing lines had 78% and 76% RRE (Figure 6C). We also observed the root morphology in all transgenic plants under Al treatment (Figures S1A and S2A). The integrity of the apical region of *Atwrky54* mutant was damaged under Al treatment, while that of Col-0 was relatively mild. The injury to the apical region of *SbWRKY22* transgenic lines (Figure S1A) or *SbWRKY65* overexpression lines (Figure S2A) was minimal. These results indicated the positive role of SbWRKY22 and SbWRKY65 in plant Al tolerance.

### 2.5. The Discovery of Downstream Genes Regulated by SbWRKY22 and SbWRKY65

**Figure 7:**
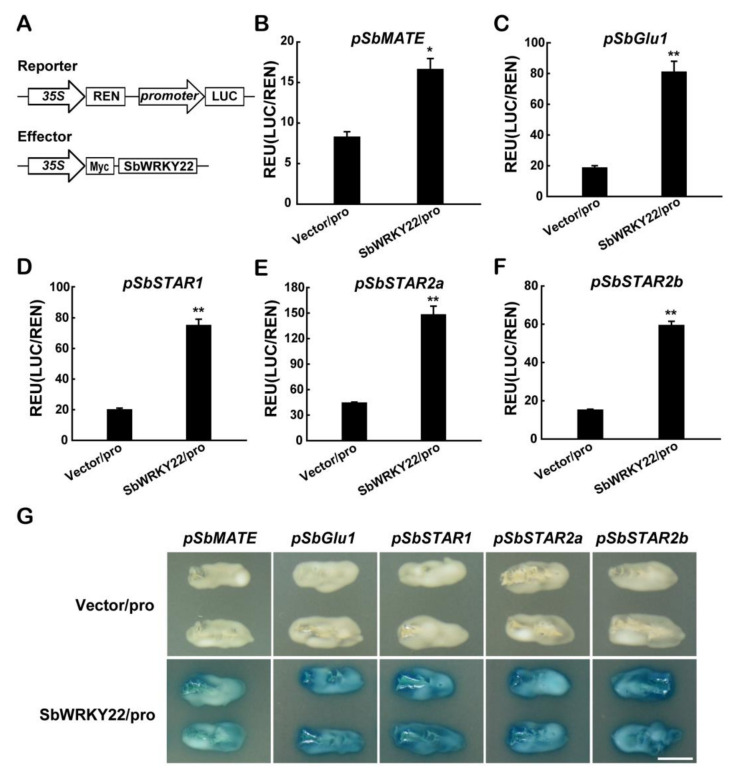
Transcriptional characteristic analysis of SbWRKY22. (**A**) Schematic diagram of the reporter and effector used in the dual-luciferase reporter system. *pSbMATE*, *SbMATE* promoter −2000 bp to −1 bp); *pSbGlu1*, *SbGlu1* promoter (−2000 bp to −1 bp); *pSbSTAR1*, *SbSTAR1* promoter (−1494 bp to −1 bp); *pSbSTAR2a*, *SbSTAR2a* promoter (−1678 bp to −1 bp); *pSbSTAR2b*, *SbSTAR2b* promoter (−1963 bp to −1 bp); LUC, firefly luciferase reporter; REN, *Renilla* luciferase reporter as an internal control; *35S*, *CaMV 35S* promoter; Myc, protein tag. (**B**–**F**) Transcriptional regulation of *SbMATE* (**B**), *SbGlu1* (**C**), *SbSTAR1* (**D**), *SbSTAR2a* (**E**), and *SbSTAR2b* (**F**) by SbWRKY22 in the dual-luciferase reporter system. Luciferase activity of the reporter (LUC) driven by the promoters (pro) was normalized to the internal control reporter (REN). (**G**) Transcriptional regulation of *SbMATE*, *SbGlu1*, *SbSTAR1*, *SbSTAR2a,* and *SbSTAR2b* by SbWRKY22 in the yeast one-hybrid system. Scale bar, 0.5 cm. Data represent the means ± SD from three independent biological replicates. Asterisk (*) represents significant differences from the vector-only control according to Dunnett’s *t* test (*p* < 0.05). Asterisks (**) represent significant differences from the vector-only control according to Dunnett’s *t* test (*p* < 0.01).

*SbMATE* (*Sb03g043890.1*) is the first gene reported to be associated with *Al tolerance* in sorghum [10]. Al tolerance genes, such as *SbGlu1 (Sb03g045630.1)*, *SbSTAR1 (Sb10g028530.1)*, *SbSTAR2a (Sb09g002000.1)*, and *SbSTAR2b (Sb09g001990.1)*, have also been discovered in sorghum in recent years [14,24]. In this study, we investigated whether these Al-tolerant genes could potentially function as downstream targets of SbWRKY22 and SbWRKY65. The *SbMATE/SbGlu1/SbSTAR1/SbSTAR2a/SbSTAR2b* promoter was individually introduced into a reporter vector to drive the firefly luciferase reporter gene (LUC). The *CaMV 35S*-promoter-driven expression of the Renilla luciferase reporter gene (REN) was applied as the internal control. The *CaMV 35S* promoter guided the insertion of *SbWRKY22* into an effector vector (Figure 7A). A dual-luciferase reporter test was conducted after co-transfecting each constructed reporter and effector into *Arabidopsis* protoplast cells. As shown in Figure 7B–F, all reporters driven by the promoters of the indicated Al-tolerant genes exhibited significantly higher luciferase activity in the presence of the SbWRKY22 effectors than the vector-only control. Furthermore, the ability of SbWRKY22 to regulate the transcript expression of the indicated Al-tolerant genes was examined in the yeast one-hybrid system. The results showed that GAD-SbWRKY22 fusion protein activated the *LacZ* reporter genes driven by the promoters of the indicated Al-tolerant genes (Figure 7G). These results demonstrated that SbWRKY22 regulates the transcription of *SbMATE*, *SbGlu1*, *SbSTAR1*, *SbSTAR2a*, and *SbSTAR2b*. The same assay was also used to evaluate the transcriptional effectiveness of SbWRKY65 for these Al-tolerant genes, and the results revealed that SbWRKY65 had few transcriptional effects on the above Al tolerance genes (Figure S3).

**Figure 8:**
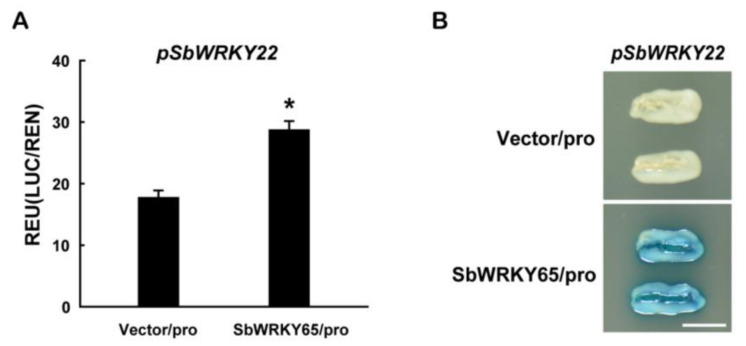
Transcriptional characteristic analysis of SbWRKY65. (**A**) Transcriptional regulation of *SbWRKY22* via SbWRKY65 in the dual-luciferase reporter system. *pSbWRKY22*, *SbWRKY22* promoter (−1680 bp to −1 bp). (**B**) Transcriptional regulation of *SbWRKY22* by SbWRKY65 in the yeast one-hybrid system. Scale bar, 0.5 cm. Data represent the means ± SD from three independent biological replicates. Asterisks (*) represent significant differences in comparison to the control according to Dunnett’s *t* test (*p* < 0.05).

WRKY transcription factors can sometimes regulate other WRKY transcription factors through cross-regulation [34]. Therefore, a dual-luciferase reporter assay was performed to investigate whether *SbWRKY22* could potentially function as the downstream targets of SbWRKY65. As shown in Figure 8A, the *SbWRKY22-*promoter-driven reporter exhibited significantly higher luciferase activity in the presence of the SbWRKY65 effector as compared to the vector-only control. Furthermore, the ability of SbWRKY65 to regulate the transcript expression of *SbWRKY22* was examined in the yeast one-hybrid system. The results showed that GAD-SbWRKY65 fusion protein activated the *LacZ* reporter gene driven by the promoter of *SbWRKY22* (Figure 8B). The above results indicated that SbWRKY65 transcriptionally regulated the expression of *SbWRKY22*. Hence, it could be speculated that SbWRKY65 may indirectly regulate the expression of the above Al-resistant genes by promoting the transcription of *SbWRKY22*.

### 2.6. Enhanced Al Tolerance Phenotype of SbWRKY22/SbWRKY65 Overexpression Plant Is Associated with Reduced Callose Deposition in Roots

**Figure 9:**
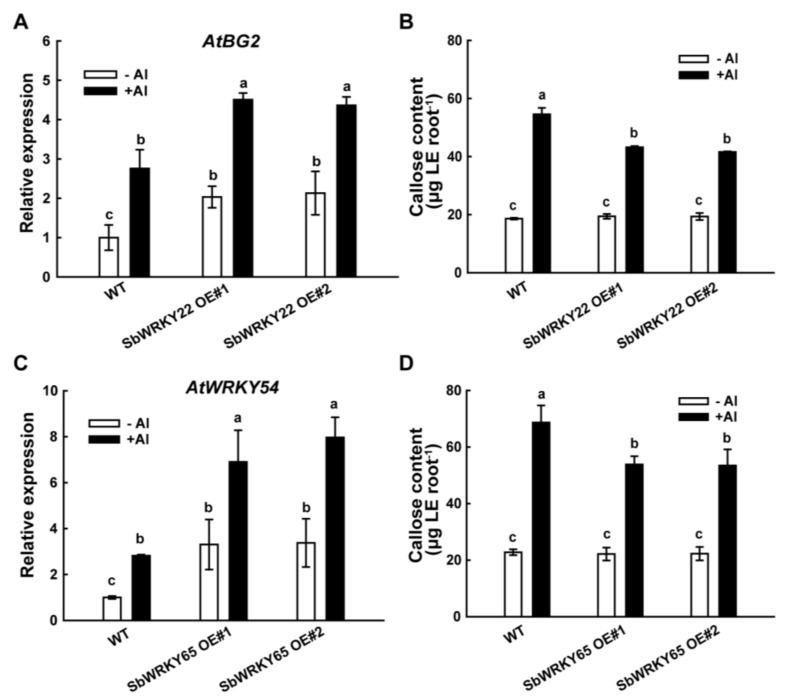
Overexpression lines of *SbWRKY22*/*SbWRKY65* show enhanced *AtBG2*/*AtWRKY54* expression and reduced callose accumulation. (**A**,**C**) Quantitative real-time PCR analysis of the expression of *AtBG2*/*AtWRKY54* in WT (Col-0) and two independent *SbWRKY22*/*SbWRKY65* overexpression lines. Seven-day-old seedlings were pre-cultured on a solid MS medium vertically and then transferred to a liquid medium containing 0.5 mM CaCl_2_ at pH 5.0 without AlCl_3_ or with 15 μM AlCl_3_ for 6 h. (**B**,**D**) Callose content in the roots of WT (Col-0) and two independent *SbWRKY22*/*SbWRKY65* overexpression lines without or with 15 μM AlCl_3_ for 6 h. Data represent the means ± SD for three independent biological replicates. Columns with different letters are significantly different according to Tukey’s test (*p* < 0.05).

Al-induced callose buildup or deposition has been utilized as a practical and quick screening parameter for Al damage as it is a sensitive indicator of Al toxicity in addition to measuring root elongation [35,36]. We previously found that the expression of the β-1,3-glucanase I gene (*SbGlu1*) was greatly up-regulated in sweet sorghum under Al stress. In the case of Al treatment, SbGlu1 efficiently lowers the buildup of callose at the root apices of sweet sorghum, improving its tolerance to Al [14]. AtBG2 (β-1,3-glucanase 2, AT3G57260.1), a homolog of SbGlu1 in *Arabidopsis*, shares 46% identity with SbGlu1. The transcript levels of *AtBG2 in WT and two independent SbWRKY22* overexpression lines were determined with or without Al stress. As shown in Figure 8A, Al induced a significant increase in *AtBG2* expression in the WT and two *SbWRKY22* overexpression lines, while on the other hand, *AtBG2* expression was significantly higher in both *SbWRKY22* overexpression lines than in the WT. This result demonstrated that SbWRKY22 was able to enhance *AtBG2* expression in *Arabidopsis*, which potentially regulates callose degradation. Therefore, callose accumulation in *SbWRKY22* transgenic lines was subsequently determined. The callose content in the roots of the WT and the overexpression lines of *SbWRKY22* was low and comparable in the absence of Al stress. However, under Al stress, the callose content in the roots of *SbWRKY22* overexpression lines was significantly lower than that in the WT (Figure 9B), suggesting that *SbWRKY22* promotes callose degradation and thereby relieves Al toxicity in plants.

Since SbWRKY65 positively regulated the expression of *SbWRKY22* (Figure 8), we assumed that SbWRKY65 may participate in some of the SbWRKY22-dependent pathways to resist Al toxicity. Thus, the transcript levels of the *SbWRKY22* homologous gene in *Arabidopsis*, *AtWRKY54*, were investigated in *SbWRKY65* overexpression lines. The results showed that SbWRKY65 was able to enhance *AtWRKY54* expression in *Arabidopsis* (Figure 9C). Meanwhile, callose accumulation in *SbWRKY65* transgenic lines with or without Al stress exhibited similar results to the *SbWRKY22* transgenic lines (Figure 9D), which verified the hypothesis that SbWRKY65 may at least participate in the SbWRKY22-dependent pathway to promote callose degradation. Callose formation in the root apex was also detected via aniline blue staining (Figures S1B and S2B), which was induced by Al and mainly distributed in the cell wall and intercellular space. *SbWRKY22* transgenic lines (Figure S1B) and *SbWRKY65* overexpression lines (Figure S2B) showed reductions in callose deposition.

All of the above results demonstrated that SbWRKY22 and SbWRKY65 could effectively reduce the accumulation of callose in plant roots when sorghum is exposed to Al stress, which has contributed to the enhanced Al tolerance phenotype of SbWRKY22/SbWRKY65 overexpression lines.

## 3. Discussion

Transcription factors are widely present in various signaling pathways in plants and are involved in the regulation of downstream functional genes. In acidic soils, Al toxicity is a major factor affecting crop yield. Transcription factors play crucial roles in regulating the expression of Al tolerance genes to cope with Al stress in plants. For instance, AtSTOP1 and OsART1, two homologous zinc finger transcription factors in *Arabidopsis* and rice, respectively, regulate the expression of a number of Al-tolerance-related genes [16,37]. Some other transcription factors involved in regulating Al-tolerance-related genes have also been discovered. AtCAMTA2 regulates the secretion of apoplastic malate in roots by activating the transcriptional expression of *AtALMT1*, thereby enhancing Al tolerance in plants [38]. By working complementarily, OsASR1 and OsASR5 regulate the transcriptional expression of *OsSTAR1*, leading to cell wall changes that reduce Al binding sites on the cell wall and increase Al tolerance in rice [39]. Sweet sorghum, a variant of grain sorghum, has a variety of strong stress resistance. However, there are only a few studies on Al tolerance in sorghum due to limited genetic transformation techniques for this species. SbSTOP1 and SbWRKY1 are the two transcription factors currently known to regulate the transcription of Al tolerance genes in sorghum, and no other transcription factors have been reported to be involved in the Al stress response [15,32]. In this study, two WRKY transcription factors, SbWRKY22 and SbWRKY65, were identified in sweet sorghum and were found to be efficient in enhancing plant tolerance to Al (Figure 5 and Figure 6).

WRKY transcription factors are one of the largest families of transcription factors in plants and are essential for regulating plant growth and development. They regulate a variety of biological processes, including responses to biotic and abiotic stresses. Based on the number of WRKY domains and the type of zinc finger motif (C_2_H_2_ or C_2_HC), SbWRKY22 belongs to group III WRKY transcription factors, while SbWRKY65 belongs to group II (Figure 1A and Figure 2A). Conserved domains in SbWRKY22 and SbWRKY65 share an identical N-terminal WRKYGQK motif but have different types of C-terminal zinc finger motifs (Figure 1A and Figure 2A). The conformation of the zinc finger structure often determines its selectivity for the target DNA sequence and its binding ability to the deep groove of the target DNA double helix [40,41]. Therefore, the difference in the type and conformation of zinc finger motifs between SbWRKY22 and SbWRKY65 may imply a difference in their target DNA, either in sequence or in conformation.

The levels of Al-induced WRKY transcription factors in plants can be positively or negatively correlated with Al toxicity. For example, in *Arabidopsis*, the expression level of *AtWRKY46* is significantly down-regulated after 3 h of Al treatment and remains almost unchanged until 24 h [27]; *OsWRKY22* responds to Al, whereby its expression level rises rapidly after Al treatment, peaks at 3 h and then rapidly decreases to its pre-Al treatment level [29]. However, research on WRKY transcription factors associated with Al stress in sorghum has been very limited thus far. In this study, both *SbWRKY22* and *SbWRKY65* displayed Al-induced expression but with distinct expression patterns during 24 h. *SbWRKY65* exhibited a quick but short response to Al stress, compared with that of *SbWRKY22* (Figure 3A). Under Al treatment, the expression level of *SbWRKY65* was significantly improved at 3 h but returned to the control level at 24 h. In contrast, the response of *SbWRKY22* to Al stress lagged behind that of *SbWRKY65*, as its response significantly increased at 6 h but continued to rise thereafter for 24 h. These differences between them in response to Al stress might reflect differences in their targets and their functional mechanisms. The expression of *SbWRKY22* and *SbWRKY65* was not only induced by Al, but also exhibited some tissue specificity, as they were mainly expressed in roots but not in shoots. These results are reasonable considering that the damage to root apices and the suppression of root extension are the initial and most visible symptoms of Al toxicity in plants [33]. Thus, both SbWRKY22 and SbWRKY65 may mainly be effective in roots. Both *SbWRKY22* overexpression lines and complemented lines showed improved Al tolerance phenotypes (Figure 5). AtWRKY65 shares the highest identity with SbWRKY65; however, the *Atwrky65* mutant did not show an Al sensitivity phenotype, which may have been due to the function redundancy in the WRKY family. Thus, only *SbWRKY65* overexpression lines were constructed, which also exhibited Al tolerance phenotypes (Figure 6).

SbWRKY22 and SbWRKY65 showed the typical characteristics of transcription factors, which are localized in the nucleus and perform transcriptional activity (Figure 4). Thus, the downstream genes regulated by them were further investigated. Recent research has demonstrated that WRKY transcription factors can affect Al tolerance in plants by regulating the expression of Al resistance genes. AtWRKY46 negatively regulates the expression of *AtALMT1* [27]. SlWRKY42 directly binds to the *cis*-element W-box in the upstream region of *SlALMT9*, leading to a decrease in its expression [28]. OsWRKY22 regulates the secretion of root citric acid mediated by *OsFRDL4*, increasing the Al tolerance in rice [29]. Certain transcription factors can also regulate numerous Al-resistant genes at once. For example, OsART1 regulates the expression of 31 genes, including *OsNrat1* and *OsSTAR1* [37]. The heterologous expression of *Glycine soja GsMAS1* in *Arabidopsis* resulted in a significant up-regulation of six Al tolerance genes, including *AtALMT1* and *AtSTOP2* [42]. AtWRKY47 regulates the expression of cell wall modification genes, *ELP* and *XTH17* [30]. In this study, we found that SbWRKY22 positively regulates the expression of *SbMATE*, *SbGlu1*, *SbSTAR1*, *SbSTAR2a*, and *SbSTAR2b* (Figure 7). These results suggest that SbWRKY22 may influence the Al tolerance of sorghum via a variety of pathways, including the secretion of citric acid, degradation of callose, and modification of cell wall components. SbWRKY65 has almost no regulatory effect on the aforementioned Al tolerance genes, while its overexpression transgenic lines still showed increased Al tolerance ability (Figure 6), implying that SbWRKY65 exerts its Al-tolerant function through additional pathways.

In *Petroselinum crispum* (Mill.) Fuss, PcWRKY1 binds to promoters of itself and PcWRKY3, demonstrating the self- and cross-regulation of WRKY transcription factors [43]. Most of the genes regulated by WRKY transcription factors contain at least two W-box elements in their promoters, which are very concentrated and can sometimes be placed in reverse. By binding to these W-boxes, WRKY transcription factors can regulate the expression of their target genes [34]. The SbWRKY22 promoter (−1680 to −1 bp) is rich in the W-box; specifically, it contains 16 W-boxes. The dual-luciferase reporter assay and the yeast one-hybrid experiment both supported the conclusion that SbWRKY65 transcriptionally regulates *SbWRKY22* (Figure 8). Thus, it could be inferred that the quick rise in *SbWRKY65* expression under Al treatment (Figure 3A) may promote the following transcription of *SbWRKY22*, ensuring sufficient SbWRKY22 to quickly regulate the transcription of downstream Al-tolerant genes. SbWRKY65 exhibited an indirect transcriptional effect on known sorghum Al tolerance genes. These findings imply that sorghum may have a very sophisticated and complex system for tolerating Al stress. Further research is required to explore whether SbWRKY65 directly regulates any additional Al-tolerant genes or regulates some unknown Al-tolerant genes. The expression of *SbWRKY65* in roots was higher or closer to that of *SbWRKY22*, providing evidence for SbWRKY65 regulating the transcription of *SbWRKY22*. The expression of *SbWRKY22* in roots (2–3 cm) was slightly induced by Al, whereas that of *SbWRKY65* was not, implying that the existence of other transcription factors regulating the expression of *SbWRKY22* besides SbWRKY65 (Figure S4).

Al-induced root apices callose accumulation is not only an indicator of Al toxicity but also a mechanism of Al toxicity in plants [44,45]. Al stress affects the transcriptional expression of a number of genes that encode enzymes to catalyze callose synthesis and degradation. The sweet sorghum gene *SbGlu1*, encoding β-1,3-glucanase to catalyze callose degradation, is overexpressed in *Arabidopsis*, thereby improving its Al tolerance via reducing callose deposition in the root [14]. The transcription factor SbSTOP1 regulates the expression of *SbGlu1*, therefore promoting the degradation of callose, leading to enhanced Al tolerance in transgenic plants [23]. These findings imply a major Al tolerance pathway in sweet sorghum via increasing the transcriptional expression of *SbGlu1* and promoting callose degradation in the root. In this study, SbWRKY22 and SbWRKY65 reduced callose accumulation in plants by up-regulating the expression of the *SbGlu1* homolog (*AtBG2*) and *SbWRKY22* homolog (*AtWRKY54*) in *Arabidopsis* under Al treatment (Figure 9). These results indicate that SbWRKY22 and SbWRKY65 could at least enhance plant Al tolerance by directly or indirectly promoting callose degradation in the root.

In summary, we identified and characterized two WRKY transcription factors, SbWRKY22 and SbWRKY65, in sweet sorghum, which are closely associated with plant Al tolerance in acidic soils. The findings of this study demonstrate that SbWRKY22 significantly up-regulates the expression of some typical Al-tolerance genes, *SbMATE*, *SbGlu1*, *SbSTAR1*, *SbSTAR2a*, and *SbSTAR2b*. The overexpression of *SbWRKY22* could reduce the accumulation of root callose, thus improving the Al tolerance of plants under Al stress. Interestingly, we also found that SbWRKY65 significantly regulates the expression of *SbWRKY22*. We speculate that SbWRKY65, via regulating the transcription of *SbWRKY22*, may indirectly regulate Al tolerance genes. Our findings reveal the function of WRKY transcription factors in regulating Al tolerance in sweet sorghum and extend our understanding of the functions and mechanisms of various WRKY transcription factor members in response to abiotic stress.

## 4. Materials and Methods

### 4.1. Plant Material and Growing Conditions

The sweet sorghum (*Sorghum bicolor* L.) cultivar POTCHETSTRM was used and cultured as described previously [15]. Briefly, seeds were surface sterilized, germinated for two days, and then were transplanted into a 0.5 mM CaCl_2_ solution (pH 4.5) for three days, followed by various treatments. The seedlings were raised in a growth chamber with a photoperiod of 14 h of light (100 µmol/m^2^ s)/10 h of darkness and a relative humidity level of 60%.

In the time-course assay, 15 root apices (0–1 cm) were excised from seedlings after the seedlings were exposed to the 0.5 mM CaCl_2_ solution (pH 4.5) containing AlCl_3_ (15 μM) for the indicated durations. The Al dose-dependent assay followed a similar procedure, except that seedlings were treated with the indicated concentrations of AlCl_3_ for 12 h. In the spatial expression assay, seedlings were exposed to a 0.5 mM CaCl_2_ solution (pH 4.5) with or without AlCl_3_ (15 μM), followed by the excision of roots (0–1 cm, 1–2 cm, or 2–3 cm) and shoots. In the metal treatment assay, the root apices (0–1 cm) of seedlings were excised after exposure to solutions of AlCl_3_ (15 μM), CuCl_2_ (0.5 μM), or LaCl_3_ (10 μM) for 12 h.

### 4.2. Sequence Analysis

The amino acid sequences of SbWRKY22/SbWRKY65 and its homologs were analyzed using BLAST in NCBI (https://www.ncbi.nlm.nih.gov/ (accessed on 26 November 2022)), the sorghum genome database (http://pgsb.helmholtzmuenchen.de/plant/sorghum/index.jsp (accessed on 9 September 2018)), and the plant transcription factor database version 4 (http://planttfdb.cbi.pku.edu.cn/ (accessed on 26 November 2022)). Sequence alignment was performed in DNASTAR and displayed in GeneDoc. The phylogenetic tree was built using the neighbor-joining approach in MEGA 7.

### 4.3. Molecular Simulation

The crystal structure of the WRKY transcription factor (PDB ID: 2AYD) was downloaded from the Protein Data Bank (http://www.rcsb.org (accessed on 18 December 2022)) for conformational analysis. The 3D structures of SbWRKY22 and SbWRKY65 were built from crystal structures of the WRKY transcription factor using the AutoDock 4.0 program and were optimized through energy minimization and molecular dynamics simulation using the program Insight II, respectively. Finally, the 3D structures of SbWRKY22 and SbWRKY65 were visually examined through Discovery studio Visualizer 3.1 (http://accelrys.com/products/discoverystudio/visualization-download.php (accessed on 18 December 2022)), and images were obtained.

### 4.4. Expression Assays

RNA isolation, reverse transcription, and quantitative real-time PCR (qRT-PCR) were carried out as described previously [14,15]. The expression of *SbWRKY22/SbWRKY65* was detected using primer pairs, as shown in Table S1. The qRT-PCR was performed using SYBR Premix ExTaq (Takara) in an Mx3005P qPCR system (Stratagene, San Diego, CA, USA) with the housekeeping gene *β-actin* (GenBank ID: X79378) as an internal control. The relative expression levels of target genes were calculated using the 2^−ΔΔCT^ approach [46].

### 4.5. Subcellular Localization Assays

The coding sequence of *SbWRKY22/SbWRKY65* was inserted into a pGWB5 vector under the control of the cauliflower mosaic virus *35S* (*CaMV 35S*) promoter via the GATEWAY recombination system using primers in Table S2. These transient plant expression vectors (*35s::GFP-SbWRKY22/SbWRKY65*) and a vector alone (*35s::GFP*) were transformed into *Arabidopsis* protoplasts that were extracted from 4-week-old plants using a PEG-mediated technique, as reported in [23]. Following this, the protoplasts were incubated in the dark for approximately 16 h at room temperature, and fluorescent images were taken under a fluorescence microscope (Axio Observer A1, Zeiss, Oberkochen, Germany).

### 4.6. Aluminum Tolerance Phenotype Analysis

The coding sequence of *SbWRKY22/SbWRKY65* was cloned into the pEGAD-GFP vector at the *Eco*RI site under the control of the *CaMV 35S* promoter (*35S::GFP- SbWRKY22/SbWRKY65*) using primers in Table S2. The vector was transformed into wild-type *Arabidopsis* (WT, Columbia-0) or an *Atwrky54* mutant using the *Agrobacterium tumefaciens*-mediated floral dip technique [47]. Transgenic seedlings were first screened with the herbicide Basta and then examined via RT-PCR. Transgenic seeds (T3) were sterilized and germinated vertically on MS medium (pH 5.8) for five days, and then, seedlings were transplanted to a medium containing 1 mM CaCl_2_ and 1% (*w*/*v*) sucrose, pH 4.5, and incubated with or without 50 μM AlCl_3_ for five days. Root growth was measured, and the relative root elongation was calculated as the ratio of elongation of roots with and without Al treatment.

### 4.7. Transfermation of DNA to Protoplasts and Dual-Luciferase Reporter Assay

The reporter plasmid and effector plasmid were constructed as described previously, with a little modification [48]. The reporter plasmid (*CaMV 35S::REN-pSbMATE/pSbGlu1/pSbSTAR1/pSbSTAR2a/pSbSTAR2b-CaMV 35S (−46)::LUC*) encodes two luciferases, of which the Renilla luciferase (REN) was controlled by the *CaMV 35S* promoter and the firefly luciferase (LUC) by the promoter indicated. *CaMV 35S* minimal promoter (−46) was synthesized [49] and inserted into the *Hin*dIII/*Bam*HI sites of the vector pGreen-0800-LUC. The effector plasmid (*CaMV 35S::Myc-SbWRKY22/SbWRKY65*) was created by cloning the DNA fragment encoding *SbWRKY22/SbWRKY65* into the vector pEGAD-Myc at the *Eco*RI site under the control of the *CaMV 35S* promoter. Primers are shown in Table S2. *Arabidopsis* protoplasts were isolated from 4-week-old plants. The protoplast/DNA mixture was incubated for 12–16 h in the dark at room temperature after the protoplasts were transformed using the PEG-mediated protoplast transformation method [23]. Finally, the transformed protoplasts were used for the dual-luciferase reporter assay according to the technical manual (Promega, E1910, Madison, WI, USA). The luminescent signal was measured with a luminometer (Berthold LB960, Bad Wildbad, Germany).

### 4.8. Yeast One-Hybrid Assay

The coding sequence of *SbWRKY22/SbWRKY65* was inserted into *Eco*RI and *Xho*I sites of a pJG4-5 vector under the control of the *GAL1* promoter, coding GAD-SbWRKY22/SbWRKY65 fusion protein (GAD, GAL4 transcriptional activation domain). The indicated promoters were inserted into *Eco*RI and *Sal*I sites of a pLacZi-2u vector to promote the expression of the LacZ reporter gene. Primers are shown in Table S2. Both constructed vectors were transformed into the yeast strain EGY48 cells. First, the co-transformation cells were plated on SD/-Leu/-Ura medium for two days, and then, they were grown on proper drop-out plates containing X-gal (5-bromo-4-chloro-3-indolyl-β-D-galactopyranoside) for blue color development [50].

### 4.9. Measurement of Callose Content in Arabidopsis Roots

Callose was extracted from *Arabidopsis* roots as described previously [23]. The callose was quantified via fluorescence spectrophotometry with an excitation wavelength of 400 nm and an emission wavelength of 500 nm, using laminarin (LE) as a standard callose source.

### 4.10. Statistical Analysis

Data collected regarding gene expression, transcriptional regulation, and callose content were the means of three replicates. The RRE of transgenic plants represent the means of more than 20 replicates. Data were analyzed for statistical comparison with IBM SPSS (version 22.0), using Tukey’s test or Dunnett’s test, where *p* < 0.05 or *p* < 0.01 as indicated was considered to be statistically significant.

## Data Availability

No new data were created or analyzed in this study. Data sharing is not applicable to this article.

## Supplementary Materials

The supporting information can be downloaded at: https://www.mdpi.com/article/10.3390/ijms241210288/s1.
