# Supplementary material for: Two Sweet Sorghum (Sorghum bicolor L.) WRKY Transcription Factors Promote Aluminum Tolerance via the Reduction in Callose Deposition
Source: Int J Mol Sci. 2023 Jun 17;24(12):10288. doi: 10.3390/ijms241210288 (PMC10298921; doi:10.3390/ijms241210288)
Supplement: Supplementary file 1 [file ijms-24-10288-s001.zip › ijms-2429964-supplementary.pdf]

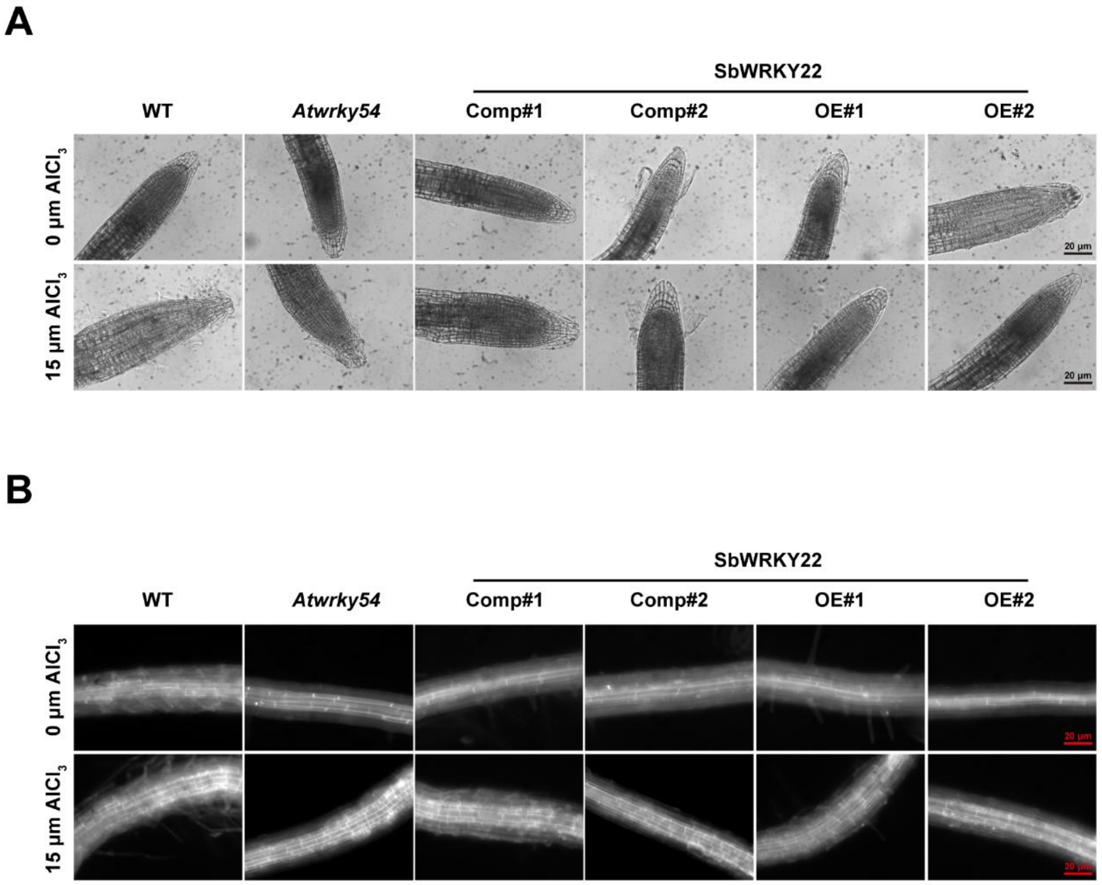

Figure S1. Effects of overexpression of *SbWRKY22* in transgenic *Arabidopsis* on root morphology and callose deposition under Al stress. **(A)** The root morphology of WT (Col-0), *Atwrky54* mutant and the transgenic lines. Seven-day-old seedlings were pre-cultured on a solid MS medium vertically, then transferred to a liquid medium containing 0.5 mM $CaCl_2$ at pH 5.0 with 15 μM $AlCl_3$ or not for 24 h. Scale bar, 20 μm. **(B)** Aniline blue staining of callose in root tips. Callose deposition in the roots of WT (Col-0), *Atwrky54* mutant and the transgenic lines with 15 μM $AlCl_3$ or not for 6 h. Callose localization was performed using root tips, stained for 5 min with an aniline blue solution consisting of 0.1% aniline blue in 1 M glycine at pH 9.5. The root tips were visualized under UV light with an inverted fluorescence microscope. Scale bar, 20 μm.

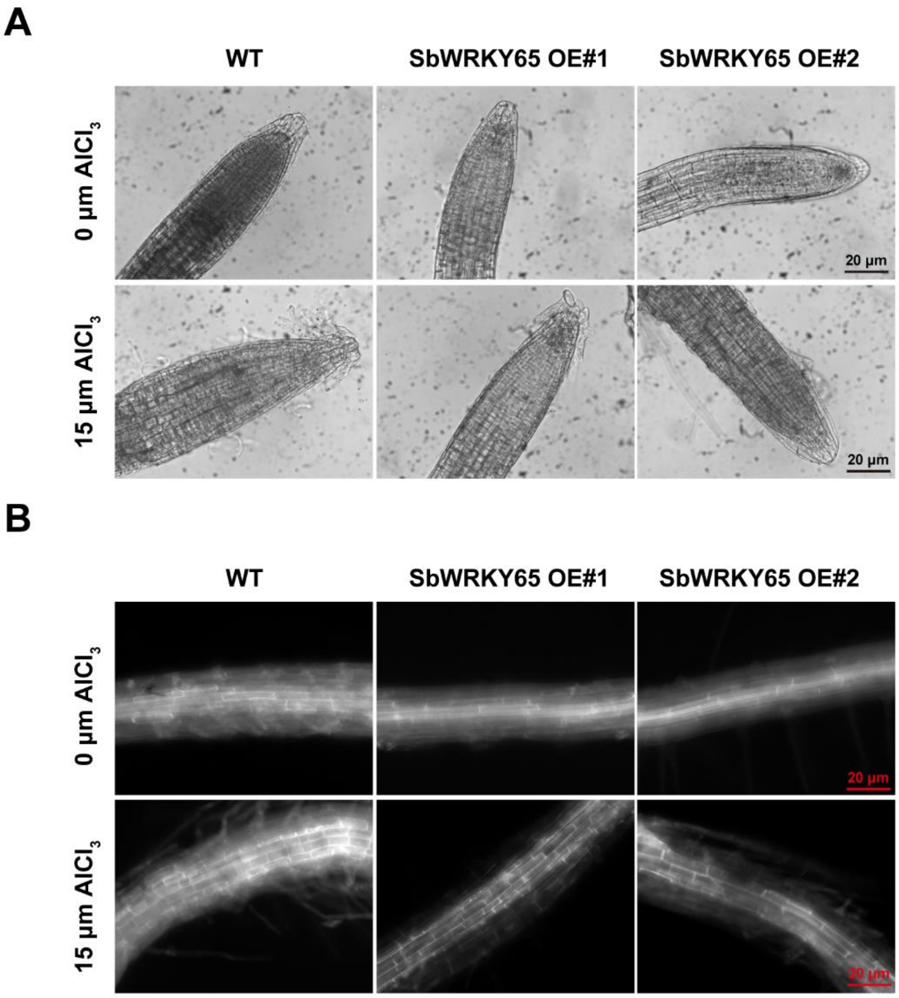

Figure S2. Effects of overexpression of *SbWRKY65* in transgenic *Arabidopsis* on root morphology and callose deposition under Al stress. **(A)** The root morphology of WT (Col-0) and two independent *SbWRKY65* overexpression lines. Seven-day-old seedlings were pre-cultured on a solid MS medium vertically, then transferred to a liquid medium containing 0.5 mM $CaCl_2$ at pH 5.0 with 15 μM $AlCl_3$ or not for 24 h. Scale bar, 20 μm. **(B)** Aniline blue staining of callose in root tips. Callose deposition patterns in the roots of WT (Col-0) and two independent *SbWRKY65* overexpression lines with 15 μM $AlCl_3$ or not for 6 h. Callose localization was performed using root tips, stained for 5 min with an aniline blue solution consisting of 0.1% aniline blue in 1 M glycine at pH 9.5. The root tips were visualized under UV light with an inverted fluorescence microscope. Scale bar, 20 μm.

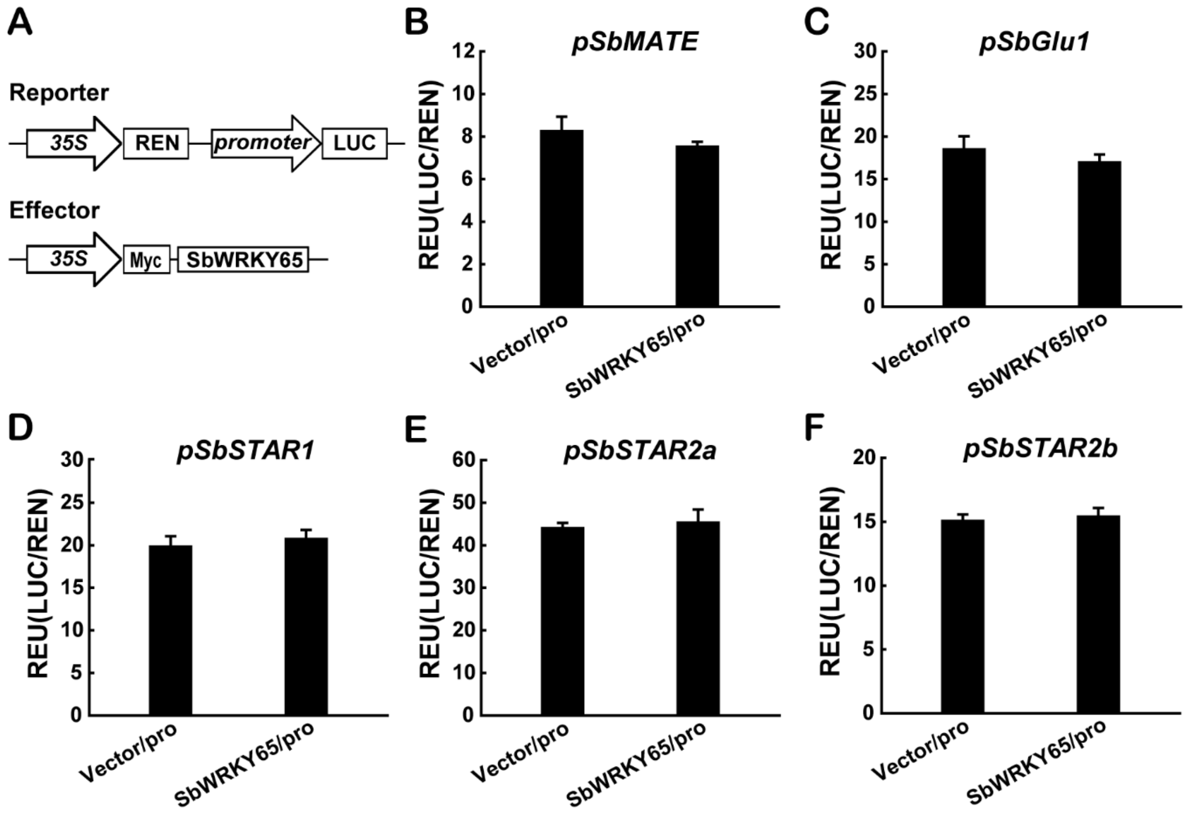

Figure S3. Transcriptional characteristic analysis of SbWRKY65. **(A)** Schematic diagram of the reporter and effector used in the dual-luciferase reporter system. *pSbMATE*, *SbMATE* promoter (-2000 bp to -1 bp); *pSbGlu1*, *SbGlu1* promoter (-2000 bp to -1 bp); *pSbSTAR1*, *SbSTAR1* promoter (-1494 bp to -1 bp); *pSbSTAR2a*, *SbSTAR2a* promoter (-1678 bp to -1 bp); *pSbSTAR2b*, *SbSTAR2b* promoter (-1963 bp to -1 bp); LUC, firefly luciferase reporter; REN, *Renilla* luciferase reporter as an internal control; *35S*, *CaMV 35S* promoter; Myc, protein tag. **(B, C, D, E, F)** Transcriptional regulation of *SbMATE* **(B)**, *SbGlu1* **(C)**, *SbSTAR1* **(D)**, *SbSTAR2a* **(E)**, and *SbSTAR2b* **(F)** by SbWRKY65. Luciferase activity of the reporter (LUC) driven by the promoters (pro) was normalized to the internal control reporter (REN). Data represent the means ± SD from three independent biological replicates.

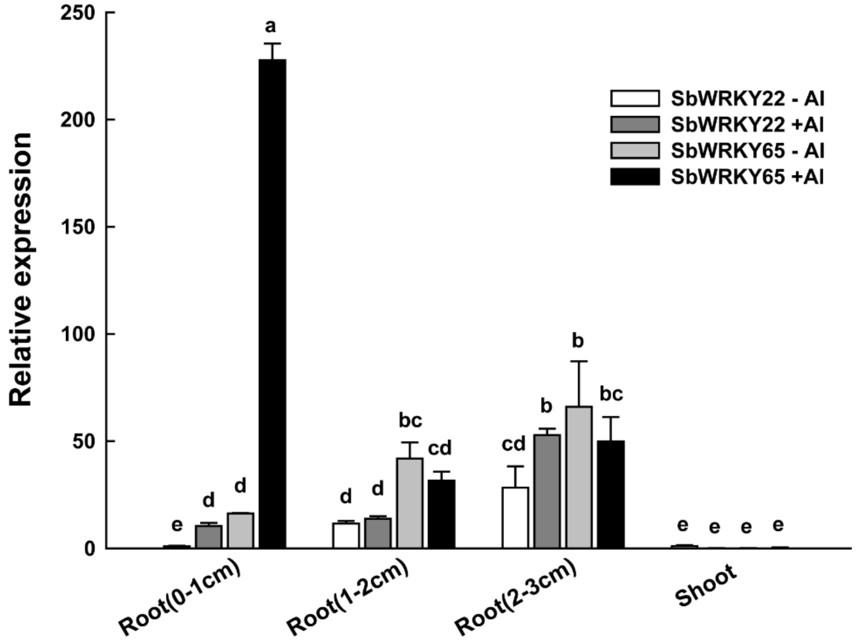

Figure S4. Relative expression of *SbWRKY22* and *SbWRKY65* in root and shoot. The expression of *SbWRKY22* in root (0-1 cm) without Al treatment was set as a unified control. Data represent the means ± SD from three independent biological replicates. Columns with different letters are significantly different according to Tukey's test (*P*<0.05).

Table S1. Sequences of primers for quantitative real-time PCR.

Gene	Primer	Sequence (5′–3′)
SbWRKY22	SbWRKY22-F	ACCACCAGTGAGCTCAGACT
	SbWRKY22-R	CTGTGCGATCTTCTCCTGGT
SbWRKY65	SbWRKY65-F	CGCCTTTTCCGAGGGCTTAC
	SbWRKY65-R	CTGTGCTCGAACGAGTAGGT
Sb-actin	Sb-actin-F	CGACCTTACCGACTACCTCATG
	Sb-actin-R	TCTTGGCAGTCTCCATCTCCT
AtBG2	AtBG2-F	CAGCTACATGGGAGACACGG
	AtBG2-R	CACGATTTCCAACGATCCGC
AtWRKY54	AtWRKY54-F	TGCACTGCCAATGACCAAAC
	AtWRKY54-R	CATGCCTGCGTCTATTGCTG
At-actin	At-actin-F	GTCTTGTTCCAGCCCTCGT
	At-actin-R	GAGATCCACATCTGCTGGAATG

Table S2. Sequences of primers for cloning of the constructs.

Primer	Sequence (5′–3′)
pGWB5-SbWRKY22-F	CAAAAAAGCAGGCTTCATGGCGTCTTCCGCTGGC
pGWB5-SbWRKY22-R	CAAGAAAGCTGGGTCTCAGGGATCGAAGCCAAACAG
pGWB5-SbWRKY65-F	CAAAAAAGCAGGCTTCATGGACGCCGAGTGGAGC
pGWB5-SbWRKY65-R	CAAGAAAGCTGGGTCTCACTTCACCCCGCCG
pEGAD-SbWRKY22-F	GGCAGCGGCCGAATTCATGGCGTCTTCCGCTGGC
pEGAD-SbWRKY22-R	CGAGCCCGGGGAATTCTCAGGGATCGAAGCCAAACAG
pEGAD-SbWRKY65-F	GGCAGCGGCCGAATTCATGGACGCCGAGTGGAGC
pEGAD-SbWRKY65-R	CGAGCCCGGGGAATTCTCACTTCACCCCGCCG
pGreen0800-pSbMATE-F	CGGTATCGATAAGCTTCTACAGACTATTAAAGTTGGTTGG
pGreen0800-pSbMATE-R	GGGTCTTGCGCCCGGGGTCGGCCTAGCTACAAACCTT
pGreen0800-pSbGlu1-F	CGGTATCGATAAGCTTGTCTGTTCCTGTATCTACTGTGTCT
pGreen0800-pSbGlu1-R	GGGTCTTGCGCCCGGGTTTGCTCGAACTCGGAGATGT
pGreen0800-pSbSTAR1-F	CGGTATCGATAAGCTTTTGAACGTTCGCTAAATTGTCTTGT
pGreen0800-pSbSTAR1-R	GGGTCTTGCGCCCGGGTATTGGCGGCGGCGG
pGreen0800-pSbSTAR2a-F	CGGTATCGATAAGCTTCATTGACCTGCAGTCCGCG
pGreen0800-pSbSTAR2a-R	GGGTCTTGCGCCCGGGGCCCGCAGCAAGCAGC
pGreen0800-pSbSTAR2b-F	CGGTATCGATAAGCTTACTTCAGGGACTGCGAAAGC
pGreen0800-pSbSTAR2b-R	GGGTCTTGCGCCCGGGCACCGACCGGCCCGTAC
pGreen0800-pSbWRKY22-F	CGGTATCGATAAGCTTGTTAAGAAATGACTTATGTTCTCC
pGreen0800-pSbWRKY22-R	GGGTCTTGCGCCCGGGGCCCGCTTGCTAGCTATCT

Table S2. Sequences of primers for cloning of the constructs (Continued).

Primer	Sequence (5′–3′)
pJG45-SbWRKY22-F	TGCCTCTCCCGAATTCATGGCGTCTTCCGCTGGC
pJG45-SbWRKY22-R	TCCAAAGCTTCTCGAGTCAGGGATCGAAGCCAAACAG
pJG45-SbWRKY65-F	TGCCTCTCCCGAATTCATGGACGCCGAGTGGAGC
pJG45-SbWRKY65-R	TCCAAAGCTTCTCGAGTCACTTCACCCCGCCG
pLacZi2u-pSbMATE-F	TATTGGATCGGAATTCCTACAGACTATTAAAGTTGGTTGG
pLacZi2u-pSbMATE-R	ATGCCTCGAGGTCGACGTCGGCCTAGCTACAAACCTT
pLacZi2u-pSbGlu1-F	TATTGGATCGGAATTCGTCTGTTCCTGTATCTACTGTGTCT
pLacZi2u-pSbGlu1-R	ATGCCTCGAGGTCGACTTTGCTCGAACTCGGAGATGT
pLacZi2u-pSbSTAR1-F	TATTGGATCGGAATTCTTGAACGTTCGCTAAATTGTCTTGT
pLacZi2u-pSbSTAR1-R	ATGCCTCGAGGTCGACTATTGGCGGCGGCGG
pLacZi2u-pSbSTAR2a-F	TATTGGATCGGAATTCCATTGACCTGCAGTCCGCG
pLacZi2u-pSbSTAR2a-R	ATGCCTCGAGGTCGACGCCCGCAGCAAGCAGC
pLacZi2u-pSbSTAR2b-F	TATTGGATCGGAATTCACTTCAGGGACTGCGAAAGC
pLacZi2u-pSbSTAR2b-R	ATGCCTCGAGGTCGACCACCGACCGGCCCGTAC
pLacZi2u-pSbWRKY22-F	TATTGGATCGGAATTCGTTAAGAAATGACTTATGTTCTCC
pLacZi2u-pSbWRKY22-R	ATGCCTCGAGGTCGACGCCCGCTTGCTAGCTATCT
